# Oxidants and antioxidants in myocardial infarction (MI): Investigation of ischemia modified albumin, malondialdehyde, superoxide dismutase and catalase in individuals diagnosed with ST elevated myocardial infarction (STEMI) and non-STEMI (NSTEMI)

**DOI:** 10.5937/jomb0-28879

**Published:** 2021-06-05

**Authors:** Nesim Aladağ, Ramazan Asoğlu, Mahmut Ozdemir, Emin Asoğlu, Atabey Rukiye Derin, Canan Demir, Halit Demir

**Affiliations:** 1 Van Yuzuncu Yil University, Faculty of Medicine, Department of Cardiology, Van, Turkey; 2 Adıyaman University, Training and Research Hospital, Cardiology Department, Adıyaman, Turkey; 3 Bayrampa a Kolan Hospital, Cardiology Department, Istanbul, Turkey; 4 Mardin Community Hospital, Cardiology Department, Mardin, Turkey; 5 University of Health Sciences, Van Training and Research Hospital, Cardiovascular Surgery Department, Van, Turkey; 6 Van Yüzüncü Yıl University, Vocational School of Health Services, Van, Turkey; 7 Van Yüzüncü Yıl University, Department of Biochemistry, Van, Turkey

**Keywords:** STEMI, NSTEMI, ischemia modified albumin, malondialdehyde acid, superoxide dismutase, catalase, STEMI, NSTEMI, albumin modifikovan ishemijom, malondialdehidna kiselina, superoksid dismutaza, katalaza

## Abstract

**Background:**

Coronary ischemia can lead to myocardial damage and necrosis. The pathogenesis of cardiovascular diseases often includes increased oxidative stress and decreased antioxidant defense. The study aimed to assess levels of ischemia modified albumin (IMA), malondialdehyde acid (MDA), superoxide dismutase (SOD), and catalase in individuals diagnosed with ST elevated myocardial infarction (STEMI) and non-STEMI.

**Methods:**

The present study prospectively included 50 STEMI patients, 55 NSTEMI patients, and 55 healthy subjects. Only patients who were recently diagnosed with STEMI or NSTEMI were included in this study. IMA, MDA, SOD, and catalase activities were measured spectrophotometrically. Significant coronary artery lesions were determined by angiography.

**Results:**

Patients with ACS had significantly greater IMA and MDA values than the healthy controls (p<0.001). Besides, patients with STEMI had IMA levels that were significantly greater than those of the patients with NSTEMI (p<0.001), while the reverse was true for MDA levels (p<0.001). The healthy controls had the highest levels of SOD and catalase levels, followed by patients with STEMI and patients with NSTEMI, respectively (p<0.001). There was a significant negative correlation among MDA and SOD with catalase levels (r = -0.771 p<0.001 MDA vs catalase; r = -0.821 p<0.001 SOD vs catalase).

**Conclusions:**

Data obtained in this study reveals that compared to healthy controls, STEMI and NSTEMI patients had increased levels of MDA and IMA and decreased levels of SOD and catalase.

## Abstract (sr)

**Uvod:**

Koronarna ishemija može dovesti do oštećenja miokarda i nekroze. Patogeneza kardiovaskularnih bolesti često uključuje povećani oksidativni stres i smanjenu anti oksidativnu odbranu. Studija je imala za cilj da proceni nivoe albumina modifikovanog ishemijom (IMA), malon dial dehidne kiseline (MDA), superoksid dismutaze (SOD) i katalaze kod osoba kojima je dijagnostikovan infarkt miokarda sa elevacijom ili podignutim ST segmentom (STEMI) i bez elevacije ST segmenta (NSTEMI).

**Metode:**

Studija je prospektivno obuhvatila 50 pacijenata sa STEMI, 55 pacijenata sa NSTEMI i 55 zdravih ispitanika. U studiju su bili uključeni samo pacijenti kojima je nedavno dijagnostikovan STEMI ili NSTEMI. IMA, MDA, SOD i aktivnosti katalaze merene su spektrofotometrijski. Značajne lezije koronarnih arterija utvrđene su angiografijom.

**Rezultati:**

Pacijenti sa ACS su imali značajno veće vrednosti IMA i MDA od zdravih ispitanika iz kontrolne grupe (p < 0,001). Pored toga, pacijenti sa STEMI su imali nivoe IMA koji su bili znatno veći od onih kod pacijenata sa NSTEMI (p < 0,001), dok je za nivoe MDA (p < 0,001) bilo obrnuto. Zdravi kontrolni ispitanici su imali najviše nivoe SOD-a i katalaze, zatim pacijenti sa STEMI i pacijenti sa NSTEMI (p < 0,001). Postojala je značajna negativna korelacija između MDA i SOD sa nivoima katalaze (r = - 0,771 p < 0,001 MDA u odnosu na katalazu; r = -0,821 p < 0,001 SOD u odnosu na katalazu).

**Zaključak:**

Podaci dobijeni u ovoj studiji otkrivaju da su u poređenju sa zdravim kontrolnim ispitanicima pacijenti sa STEMI i NSTEMI imali povišene nivoe MDA i IMA i smanjene nivoe SOD i katalaze.

## Introduction

Acute coronary syndrome (ACS) is diagnosed by the co-evaluation of typical chest pain, electrocardiography (ECG) changes, and ischemia biomarkers, such as troponin and creatine kinase-MB (CKMB). Failure to diagnose ACS upon presentation to the emergency room could result in adverse cardiac events in patients with cardiac ischemia [1]. Therefore, cardiac biomarkers may be useful in patients with atypical presentation and no changes in ECG. Because troponin does not increase in the early stages of cardiac ischemia and myocardial necrosis, biomarkers such as myoglobin and ischemia modified albumin (IMA) should be investigated in ACS diagnosis. IMA is a new biomarker for the detection of myocardial ischemia. IMA cannot bind transition metals such as cobalt. Also, IMA levels increase within minutes after ischemia begins, remain high for 6-12 hours, and revert to normal within 24 hours [2].

Cardiac ischemia causes oxidative stress, leading to the generation of reactive oxygen species (ROS), which in turn causes modifications to cellular structures (e.g., proteins and nucleic acids) [3]. The malondialdehyde acid (MDA), which is a marker of lipid peroxidation, is generated when oxygen-derived free radicals surround and break-down polyunsaturated fatty acids [4]. MDA is a known oxidative stress marker for coronary artery disease (CAD) severity and plaque sensitivity [5] [6]. Moreover, MDA levels are significantly higher in ACS patients than in healthy controls, and they are inversely correlated with antioxidant parameters in ACS patients [7]. Additionally, Nand et al. [8] reported that MDA levels increased three-fold in acute myocardial infarction (AMI) patients compared to a control group.

It is known that increased oxidative stress and decreased antioxidant defense play major roles in the pathogenesis of cardiovascular diseases [9]. Superoxide dismutase (SOD) and catalase protect cells against oxidative stress. SOD converts superoxide radicals to hydrogen peroxide (H_2_O_2_), which is then reduced to water and oxygen by the catalase enzyme system [10]. It has been reported that SOD activity and catalase levels are decreased in AMI patients [11] [12]. When the clinical situation in ACS patients progresses from unstable angina (UA) to STEMI, oxidative stress increases, while antioxidant status decreases [13]. In addition, Gammoudi et al. [14] reported that SOD activity decreases with increased oxidative stress.

Because ACS is known to be accompanied by increased oxidative stress and decreased antioxidant levels, the aim of the current study was to elucidate the oxidative, and antioxidant status of ST elevated myocardial infarction (STEMI) and non-STEMI (NSTEMI) patients by evaluating IMA, MDA, SOD and catalase levels. As far as we know, this is the first study to investigate the IMA, MDA, SOD, and catalase biomarkers instantly in ACS patients.

## Materials and Methods

This prospective study includes 50 STEMI patients and 55 NSTEMI patients who applied to the emergency service of Van Training and Research Hospital with chest pain between October 2018 and October 2019. The convenience sampling method was used in this study. The control group included 55 healthy volunteers with no history of coronary artery disease. A cardiologist examined all study participants and recorded their medical histories. All of the enrolled patients underwent ECG within one hour of admission. Patients were included in this study if they had been recently diagnosed with STEMI or NSTEMI at presentation. Patients were excluded from this study if they were <18 years of age, had an acute or chronic infection, had pulmonary embolism, had stable or unstable angina pectoris, had systolic heart failure (<40% ejection fraction), had a severe valvular disease, had tachyarrhythmia, had coronary artery bypass graft surgery, were pregnant, had end-stage renal insufficiency, had hematological disorders, had a malignant disease, had a cerebrovascular stroke, had chronic lung disease, had renal or hepatic dysfunction, had a systemic infection, and/or had inflammatory diseases.

Patients with ACS were separated into two groups (STEMI and NSTEMI). Of the included patients, 50 were identified as STEMI. The STEMI diagnosis was made after the evaluation of clinical symptoms, and if the patient had an elevation of ST in 2 leads by ECG, had an obstruction in the main branch of a coronary artery by coronary angiography, and had significantly increased levels of serum troponin-I (≥2x the normal upper limit) and CKMB. The other 55 included patients were identified as NSTE-MI. The NSTEMI diagnosis was made after evaluating clinical symptoms, and if the patient had marked T wave inversion or ST-segment depression by ECG, and if the patient had elevated concentrations of myocardial necrosis biomarkers. STEMI patients were given 180 mg ticagrelor/60 mg prasugrel/600 mg clopidogrel, 300 mg aspirin, and intravenous heparin (70-100 U/kg) prior to undergoing primary percutaneous coronary intervention (PCI). Following PCI, the STEMI patients were given beta-blockers and statins. NSTEMI patients were treated with 300 mg aspirin, a loading dose of 300 mg clopidogrel (followed by 75 mg), low-molecular-weight heparin, statins, and beta-blockers at admission. Angiography was performed within three days in NSTEMI patients.

Demographic data, cardiovascular risk factors (including body mass index (BMI, kg/m^2^), and medical history were recorded at admission. Each included patient provided informed consent prior to the start of the study. This study was performed in accordance with the standards stated in the Declaration of Helsinki and was approved by the Van Training and Research Hospital Ethics Committee. A venous blood sample was taken from each patient within twelve hours of the onset of symptoms, and this blood sample was analyzed for CK-MB, troponin I, low-density lipoprotein-cholesterol (LDL), creatinine, and whole blood count (WBC). Troponin and CK-MB levels were measured using Siemens ADVIA Centaur Cp analyzers in the emergency laboratory.

Venous blood was spun down (5000 RPM, 10 min) and frozen (-80°C) until it was analyzed for IMA via an albumin cobalt binding test results recorded in absorbance units (ABSU) [15]. High-performance liquid chromatography (HPLC, Shimadzu LC-10AT) was used to determine serum levels of MDA (method developed by Young and Trimble (inter-assay CV 6.8%, intra-assay CV 4.2%) [16]. The activity of SOD (EC 1.15.1.1) was identified spectrophotometrically [17]. In this method, the oxidation of NADPH by superoxide radicals is monitored (by absorbance at 540 nm). The oxidation of NADPH (in the presence of mercaptoethanol, MnCl_2_, and EDTA) results in the production of a superoxide anion (O_2_^-^) from molecular oxygen. When samples containing SOD (plasma or hemolysate) are added to the reaction mixture, the oxidation of NADPH is proportionately inhibited. Bovine erythrocytic SOD was run as a standard for each plate. Finally, serum activity of CAT was measured as described by Goth (inter-assay CV 4.5%, intra-assay CV 3.8%) [18].

### Statistical Analyses

All of the included data in this study were analyzed using SPSS software (v. 25.0 for Windows (SPSS Inc, Chicago, Illinois). Whether or not the continuous variables were normally distributed was determined via a Kolmogorov-Smirnov test. Variables that did have a normal distribution are presented as means±standard deviation (SD), while those that did not have a normal distribution are presented as medians with interquartile range (IQR). All of the categorical variables in this study are listed as percentages. Regarding multiple comparisons, data that had a normal distribution and was continuous were analyzed via a one-way analysis of variance (ANOVA) test followed by a Tukey post hoc test, while continuous data that were not normally distributed were analyzed with a nonparametric Kruskal-Wallis test. Fisher's exact test or a chi-square test were used to determine the frequencies of nominal variables. Pearson's or Spearman's tests were applied to analyze correlations between any of the study's continuous variables. Values of p<0.05 were defined as significant.

## Results

All of the patient and control group clinical and demographic data is presented in Table 1.

**Table 1:** Clinical and demographic data of the included patients NSTEMI, non-ST elevated myocardial infarction; STEMI, ST elevated myocardial infarction; BMI, body mass index, LDL, low-density lipoprotein, CKMB, creatine kinas-MB; WBC, whole blood cell count; IMA, ischemia modified albumin; MDA, malondialdehyde; SOD, superoxide dismutase. ^a^: Control vs. other groups. ^b^: NSTEMI vs. STEMI.

	Control (n=55)	NSTEMI (n=55)	STEMI (n=50)	p
Age (years)	55.9±9.3	59.6±12.6	58.0±14.1	0.09
BMI (kg/m^2^)	25.5±2.6	25.9±3.3	25.9±3.2	0.29
Male/Female n	29/26	37/18	31/11	0.08
Smoking n (%)	9(16)	20(36)^a^	17(34)^a^	0.01
Diabetes mellitus n (%)	14(25)	23(42)	19(45)	0.08
Hypertension n (%)	15(27)	24(44)	20(40)	0.07
Hyperlipidemia n (%)	3(5)	9(16)	1(2)	0.06
Creatinine (mmol/L)	12.61±3.6	16.22±5.41^a^	14.41±3.60^a^	0.001
LDL (mmol/L)	2081.08±531.53	1812.61±679.28	2039.64±656.31	0.05
CKMB (mmol/L)	232.43±86.49	792(522–1405)^a^	270(594–2162)^a,b^	<0.001
Troponin (mmol/L)	0.288±0.09	66.67(28–396)^a^	374.77±133.33^a,b^	<0.001
WBC (10^3^/mm^3^)	7.2±1.9	10.3±4.2^a^	9.8±4.2^a,b^	<0.001
Hemoglobin (g/L)	132±20	143±17^a^	143±16^a^	0.002
Platelet count (10^3^/mm^3^)	262.7±39.5	239.0±71.6	237.1±58.4	0.05
IMA (U/L)	900±100	180±300^a^	2400±100^a,b^	<0.001
MDA (mmol/L)	1490±30	3140±60^a^	2800±1100^a,b^	<0.001
SOD (U/L)	2310±20	1100±30^a^	1480±40^a,b ^	<0.001
Catalase (U/L)	540±20	220±20^a^	290±10^a,b^	<0.001
1 vessel n (%)		35(63)	35(83)	0.03
2 vessels n (%)		12(21)	4(10)	0.16
3 vessels n (%)		8(16)	3(7)	0.17

Patients with STEMI and NSTEMI were significantly older than those in the control group. Members of the STEMI and NSTEMI groups smoked significantly more than the subjects in the control group (p=0.01). Regarding cardiac ischemia parameters, troponin levels were highest in the STEMI group and lowest in healthy controls (p<0.001). In contrast, CKMB levels were significantly higher in the NSTEMI group when compared to STEMI patients and the control group (p<0.001).

**Figure 1:**
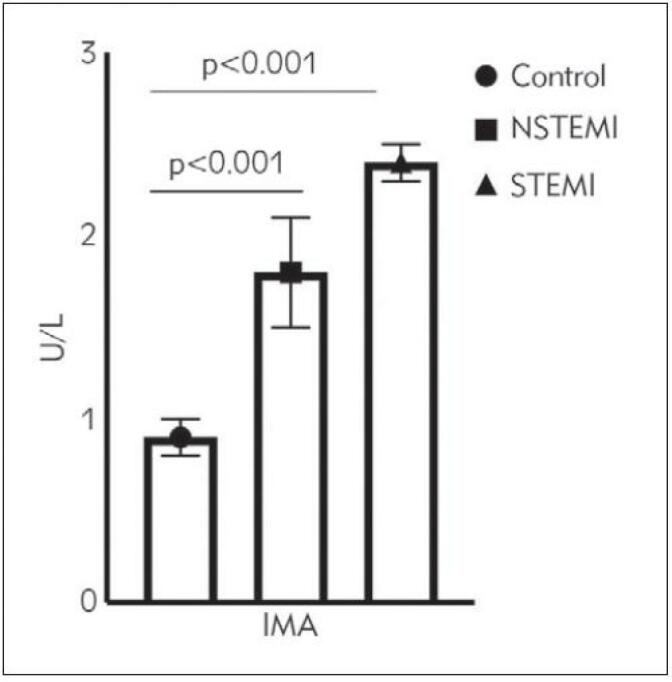
Ischemia modified albumin (IMA) levels betweenstudy groups

**Figure 2:**
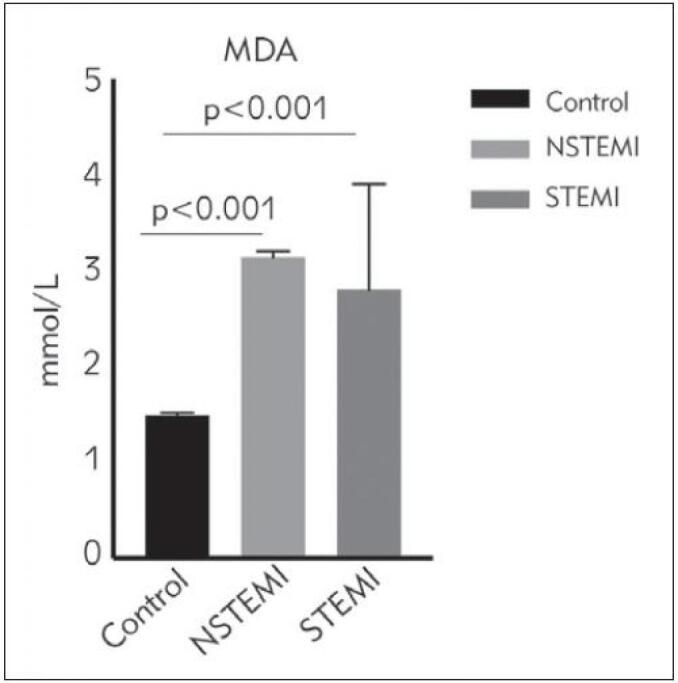
Malondialdehyde (MDA) levels between study groups

**Figure 3:**
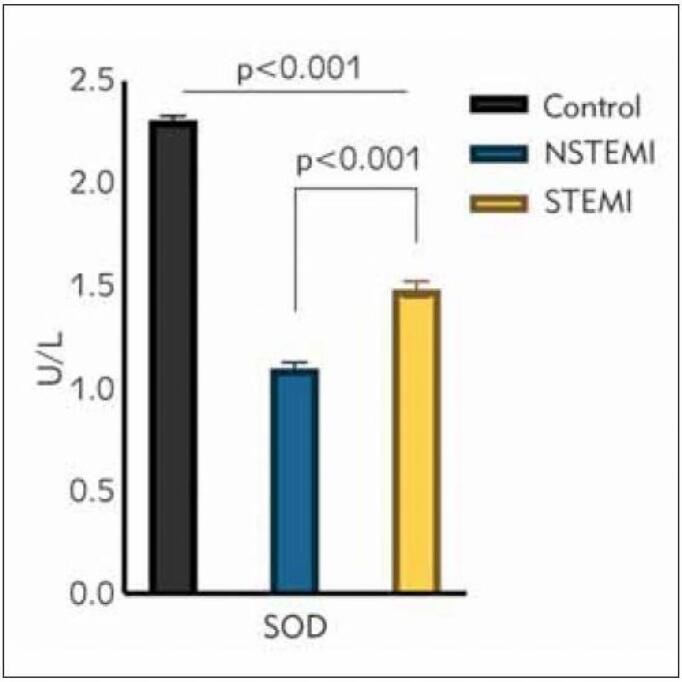
Superoxide dismutase (SOD) levels between study groups

The IMA levels of ACS patients were significantly higher than those of healthy controls (p<0.001), while those of patients with STEMI were significantly higher than those of the NSTEMI group (2.4±0.1 STEMI vs. 1.8±0.3 NSTEMI; p<0.001). The IMA values of each group are presented in Figure 1. Levels of the oxidative stress parameter, MDA, were significantly elevated in patients with ACS compared to the MDA levels of the control group (p<0.001). In addition, the NSTEMI group's MDA levels were significantly higher than those of patients with STEMI (3.14±0.06 NSTEMI vs. 2.80±1.10 STEMI; p<0.001) (Figure 2). Furthermore, the oxidative stress parameter, SOD, had its highest levels in the control group, followed by the STEMI and NSTEMI groups (2.31±0.02 controls, 1.48±0.04 STEMI, 1.10±0.03 NSTEMI; p<0.001). The SOD levels of the control group were significantly higher than those of the ACS groups, and importantly, the SOD values of the STEMI patients were significantly higher than those of the NSTEMI patients. The SOD levels of the STEMI, NSTEMI, and control groups are shown in (Figure 3).

**Figure 4:**
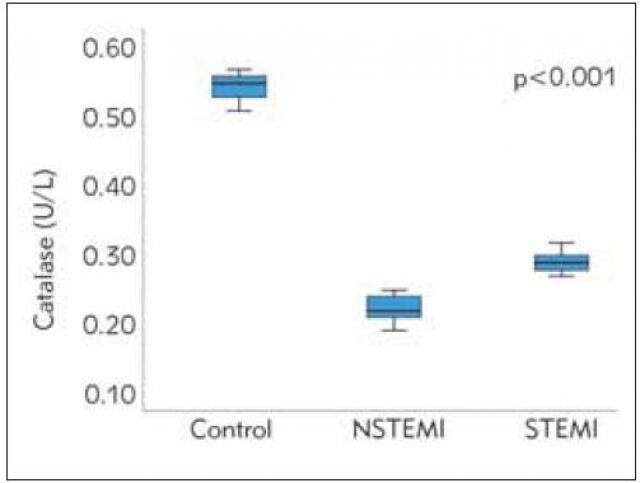
Catalase levels between study groups

Catalase levels were highest in healthy subjects in comparison with the ACS patients, while STEMI patients had significantly higher catalase levels than NSTEMI patients (0.54±0.02 controls, 0.29±0.01 STEMI, 0.22±0.02 NSTEMI; p<0.001) (Figure 4).

**Figure 5:**
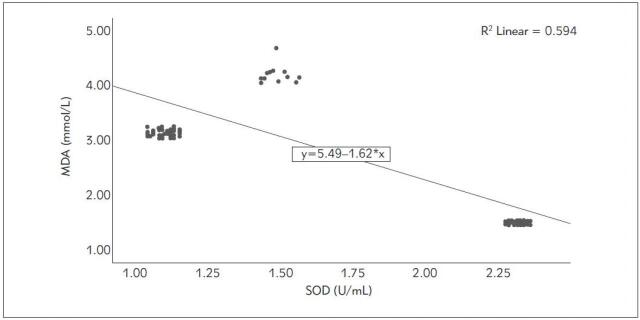
The correlation analyzes between MDA (malondialdehyde) and SOD (superoxide dismutase) levels

**Figure 6:**
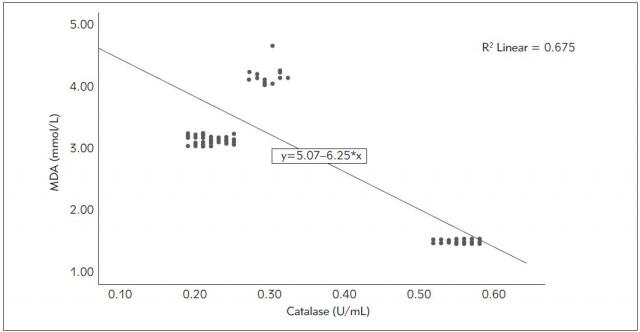
The correlation analyzes between MDA (malondialdehyde) and catalase levels

The correlation analyses among IMA, MDA, SOD, catalase, and troponin levels are presented in Table 2. The analyses indicated a positive correlation between IMA levels and MDA and troponin, and an inverse correlation with SOD and catalase. MDA and troponin had a positive correlation (r= 0.742 p<0.001), while MDA and SOD were inversely correlated (r=-0.771 p<0.001). Figure 5 shows the correlation analyses between MDA and SOD levels. There was a significant negative correlation between MDA values and catalase levels (r=-0.821 p<0.001). Figure 6 shows the correlation analyses between MDA and catalase levels. Furthermore, SOD levels had a positive correlation with catalase and an inverse correlation with troponin (r= 0.986 p<0.001; r=-0.483 p<0.001, respectively). Further more, there was a significant correlation between catalase levels and troponin levels (r=-0.544 p<0.001).

**Table 2:** Correlation analyzes among the IMA (ischemia modified albumin), MDA (malondialdehyde), SOD (superoxide dismutase), catalase, and troponin

	IMA		MDA		SOD		Catalase		Troponin	
	r	p	r	p	r	p	r	p	r	p
IMA	–	–	0.956	<0.001	-0.721	<0.001	-0.772	<0.001	0.721	<0.001
MDA	0.956	<0.001	–	–	-0.771	<0.001	-0.821	<0.001	0.742	<0.001
SOD	-0.721	<0.001	-0.771	<0.001	–	–	0.986	<0.001	-0.483	<0.001
Catalase	-0.772	<0.001	-0.821	<0.001	0.986	<0.001	–	–	-0.544	<0.001
Troponin	0.721	<0.001	0.742	<0.001	-0.483	<0.001	-0.544	<0.001	–	–

## Discussion

Data herein indicate that ACS patients have significantly higher MDA levels than healthy controls. On the other hand, SOD and catalase, which play essential roles in antioxidant defense, were significantly higher in the control group than in ACS patients. These findings reveal that STEMI and NSTEMI patients have high oxidative stress and low antioxidant defense.

IMA is modified human albumin with impaired copper binding capacity (due to ROS) that is produced by oxidative stress [19]. IMA levels increase with cell or endothelial hypoxia, ROS, and acidosis [20]. IMA is a cardiac biomarker that is used to diagnose myocardial ischemia before necrosis in the heart tissue. In the case of cardiac ischemia, IMA is rapidly released into the bloodstream and can be measured for 24 hours. In the current study, IMA levels were the highest in patients with STEMI (vs control and patients with NSTEMI). Therefore, we conclude that patients with STEMI have a more ischemic condition than patients with NSTEMI. Sinha et al. [21] reported that IMA levels in STEMI increase due to thrombus, which restricts coronary blood flow. The authors of that study also reported that IMA levels were more sensitive in diagnosing STEMI than the combination of ECG and troponin [21]. Moreover, Liyan et al. [22] reported that individuals with ACS had higher levels of IMA than patients with non-ischemic chest pain. That same study also indicated that IMA is a sensitive biomarker for diagnosing ACS with heart-type fatty acidbinding protein [22]. The current study results are in congruence with the results reported by Aggarwal et al. [23], in that both studies indicate a significant correlation between IMA and troponin levels. Moreover, IMA levels have been associated with short and longterm recurrent ischemia; IMA has a higher prognostic value than troponin [24]. Furthermore, it has been reported that IMA levels are independent predictors for one-year cardiac adverse events in both STEMI and NSTEMI patients [25].

MDA is a biomarker used to measure lipid peroxidation, which is often associated with cardiovascular disease [4]. The measurement and follow-up of lipid peroxidation levels was proposed in a previous study regarding the diagnosis and treatment of ACS patients [26]. Higher MDA levels are related to ischemic damage or unstable plaque in ACS patients [27]. Moreover, MDA levels were correlated with the AMI rate, and MDA levels have been used as a determinant for severe coronary diseases [28]. Nand et al. reported that MDA levels in an AMI group increased three-fold compared to those of healthy individuals [8]. The current study revealed that levels of MDA were significantly higher in patients with ACS as compared to healthy individuals; furthermore, MDA levels were greater in patients with NSTEMI versus those with STEMI. Taken together, these data indicate that oxidative stress is high in ACS patients. The study by Serdar et al. [13] evaluated both the oxidant and antioxidant status of UA, NSTEMI, and STEMI patients. Results of that study indicate that there was in increase in oxidative stress increased, and a decrease in antioxidant status in patients as they progress from a UA diagnosis to a STEMI diagnosis. On the contrary, results of the current study found that NSTEMI and STEMI patients had similar levels of MDA. In accordance with the results of the current study, Holvoet et al. [27] reported that there was no difference between MDA levels in UA versus AMI patients.

Antioxidant mechanisms prevent the harmful effects of free radicals. SOD is an enzyme involved in antioxidant defense that reduces the superoxide radical to hydrogen peroxide and oxygen. Thus, it plays a vital role in the control of superoxide levels in tissues.

It is known that SOD levels are linked with increased oxidative stress in coronary artery disease (CAD). One publication indicated that the levels of SOD in patients with ACS were lower than those of a control group [29]. Further, Gammoudi et al. stated that reduced SOD levels in CAD patients were linked to increased oxidative stress [14]. The present study results indicate that ACS patients have significantly lower SOD levels than the healthy controls, with NSTEMI patients having lower levels than STEMI patients. These findings indicate that NSTEMI patients had higher severe oxidative stress and lower antioxidant enzyme levels. Similar to what has been previously reported, data from this study also indicate that patients with ACS have significantly lower levels of SOD than healthy controls. On the other hand, Himmetoglu et al. [30] reported that there were no differences in levels of SOD in AMI patients versus healthy controls. We hypothesize that acute ischemia could be related to an increase in free radical production and a corresponding depletion of antioxidant defenses. The results of the current study suggest that decreased SOD and catalase levels might be due to increased utilization to trap the free radicals induced by myocardial ischemia.

Catalase is important for maintaining the balance between oxygen and hydrogen peroxide. Catalase breaks down hydrogen peroxide into two molecules; water and oxygen. A previous study revealed that catalase protects cells against ROS [31]. In the present study, catalase levels of ACS patients were than those of the healthy controls, with levels being significantly lower in NSTEMI versus STEMI patients. These findings indicate that NSTEMI patients had a lower enzymatic antioxidant defense compared to STEMI patients. Bagatini et al. [32] reported that AMI patients had higher oxidative stress, lower non-enzymatic antioxidant parameters (Vitamin C and Vitamin A), and higher enzymatic antioxidant parameters (SOD and catalase) than healthy controls. In that study, the fact that AMI patients had higher SOD and catalase levels may be because these parameters were analyzed when the troponin levels were highest (average 72 hours after AMI). In addition, a previous study showed that catalase and SOD increase in AMI to mitigate any tissue damage due to oxidative stress [33].

On the other hand, Senthil et al. [34] reported that AMI patients had decreased SOD and catalase levels. The discrepancies in the results of the various studies may be due to the fact that there is an initial decrease in antioxidant enzyme levels, followed by an increase in their levels after ROS generation, such as in myocardial injury followed by reperfusion [35]. Furthermore, Serdar et al. [13] reported that vitamins and antioxidant enzymes were significantly de creased, and oxidative markers were significantly increased in CAD patients. The authors stated that decreased antioxidant enzyme levels are due to increased oxida-tive stress in CAD patients. The same study also showed that increased oxidative stress is associated with severe CAD [36].

### Limitations

One of the limitations of the current study is that the study group consisted solely of ACS patients with STEMI and NSTEMI. Hence, our findings are not generalizable for patients with UA and stable angina pectoris. The relatively limited number of patients could limit the strength of results and conclusions obtained from this study. In addition, although the IMA, MDA, SOD, and catalase activities were measured within six hours of admission, we did not perform serial measurements of these parameters. Another limitation may be that oxidative, and antioxidant enzymes were not compared with different ischemia markers, such as myoglobin. Further, nonenzymatic antioxidant levels, such as those of vitamins C and A, were not analyzed in our study. Future investigations with a greater sample size are needed to evaluate the IMA, MDA, SOD, and catalase measurement for the evaluation of patients presented with STEMI and NSTEMI.

## Conclusions

In conclusion, data from the current study reveals that levels of MDA and IMA were significantly increased, and SOD and catalase activities were significantly decreased in patients with STEMI and NSTEMI, suggesting that STEMI and NSTEMI patients are in a state of heightened oxidative stress. Understanding the relationship of the potential usefulness of blood concentrations of oxidant and antioxidant parameters with ACS requires further study.

## Acknowledgments

This study received no grant from any funding agency in public, commercial, or not-for-profit sectors.

## Conflict of interest statement

All the authors declare that they have no conflict of interest in this work.

## List of abbreviations

IMA, ischemia modified albumin; ROS, reactive oxygen species; MDA, malondialdehyde acid; VSA, vasospastic angina; AMI, acute myocardial infarction; SOD, superoxide dismutase.
